# Drug Metabolism and Pharmacokinetic Evaluation of a Novel RNase H2 Inhibitor for the Treatment of Triple-Negative Breast Cancer

**DOI:** 10.3390/pharmaceutics17081052

**Published:** 2025-08-13

**Authors:** Yang Wang, Huan Xie, Jing Ma, Ting Du, Song Gao, Yuan Chen, Shiaw-Yih Lin, Dong Liang

**Affiliations:** 1 Department of Pharmaceutical Science, College of Pharmacy and Health Sciences, Texas Southern University, Houston, TX 77004-9987, USAhuan.xie@tsu.edu (H.X.); song.gao@tsu.edu (S.G.); yuan.chen@tsu.edu (Y.C.); 2 Department of Systems Biology, MD Anderson Cancer Center, Houston, TX 77030-1515, USA

**Keywords:** RNase H2 inhibitor R14, drug metabolism, pharmacokinetic evaluation

## Abstract

**Objectives:** A thorough understanding of pharmacokinetics and metabolism is critical during early drug development. This study investigates the absorption, distribution, metabolism, and excretion (ADME) profile of R14, a novel compound, using a combination of in vitro and in vivo approaches. **Methods:** In vitro studies included Caco-2 permeability assays, metabolic stability evaluations in liver microsomes and hepatocytes, and identification of CYP isoforms responsible for R14 metabolism. In vivo pharmacokinetic and metabolic profiling was conducted in rats following oral administration. R14 was quantified using UHPLC-MS/MS. Metabolites were identified using high-resolution UHPLC- QTOF MS/MS, and relative exposure was estimated using peak area-derived AUCs. **Results:** R14 exhibited low oral bioavailability (13.4%) and high systemic clearance (2.63 L/h/kg), indicating high hepatic extraction. A total of 21 plasma and 38 urine metabolites were identified. Major metabolic pathways included initial hydroxylation and hydrogenation, followed by sequential methylation and Phase II conjugations (glucuronidation and sulfation). Key metabolites (M3, M4, M22, M38) accounted for the majority of systemic exposure. Less than 1% of the unchanged drug was excreted in urine, confirming extensive metabolism. Notably, discrepancies between in vitro and in vivo metabolite profiles suggested rapid further transformation of initial metabolites in vivo, which were not fully captured in vitro. **Conclusions:** This study demonstrates an efficient and integrated strategy for early-phase ADME characterization. The combined use of in vitro assays and in vivo studies, guided by advanced analytical techniques, provides a robust framework for understanding drug metabolism. These findings can inform drug optimization and help minimize risks in later stages of development.

## 1. Introduction

Triple-negative breast cancer (TNBC) is an aggressive type of invasive breast cancer and often occurs in young women. TNBC is a highly aggressive cancer type, characterized by the widespread occurrence of metastases at diagnosis and therapeutic resistance. For metastatic TNBC, the 5-year survival rate is only 10–15% [1,2]. TNBC has fewer treatment options than other types of invasive breast cancer [3]. This is because the cancer cells do not have the estrogen or progesterone receptors or enough of the HER2 protein to make hormone therapy or targeted HER2 drugs work. Thus, there is an urgent need to search for novel therapy to treat TNBC.

Recent studies have found that ribonuclease H2 (RNase H2) is overexpressed in TNBC tumors and cell lines, correlating with poor survival across multiple patient cohorts. We identified a potent RNase H2 inhibitor, R14, that has superior efficacy against xenograft TNBC mice models. We found that oral R14 administration significantly reduced tumor size and improved survival in MDA-MB-231 tumor-bearing mice compared to vehicle controls [4].

R14, also known as THr101 [5] or 6-Fluoro-2-(2-methylphenyl)-1,2-benzothiazol-3(2H)-one, is an aryl lactam compound that is poorly water soluble and has limited solubility in DMSO (25 mg/mL). In vivo, R14 was rapidly absorbed after oral administration to mice and showed a bi-exponential disposition profile [6]. R14 was found to completely disappear from mouse whole blood samples within 10 days after stored at −70 °C. Pharmacokinetic plasma samples containing R14 were successfully stabilized using citric acid and sodium sulfite for ultra-high-performance liquid chromatography with tandem mass spectrometry (UHPLC-MS/MS) quantifications [6]. The superior anti-TNBC efficacy in mice models and observed limited drug accumulation in major organs warranted further preclinical drug development exploration of R14 as a drug candidate.

The identification of unknown metabolites and their metabolic pathways is a critical component of preclinical drug development, enabling more accurate predictions of a drug’s disposition and pharmacodynamic effects in humans [7,8]. Early metabolite characterization offers valuable insights into the drug’s fate, supporting the prediction of efficacy, toxicity, and potential drug–drug interactions (DDIs). Such information is vital for selecting and optimizing drug candidates, ultimately helping to reduce attrition rates and overall development costs [9].

The U.S. Food and Drug Administration (FDA) recommends metabolite profiling for any metabolite that accounts for more than 10% of the total drug-related exposure in circulation [10]. Traditionally, metabolite identification has relied on radioactive labeling, extensive biological sample collection, and time-consuming chromatographic separation—methods that are labor-intensive and resource-demanding. However, the emergence of high-resolution (HR) mass spectrometry (MS) has significantly transformed this landscape. By offering enhanced accuracy, sensitivity, and throughput, HR MS has greatly improved the efficiency and reliability of metabolite identification, thereby streamlining the drug development process and strengthening the assessment of metabolic safety risks.

Sprague Dawley (SD) rats are widely used in drug development due to their well-characterized physiology, ease of handling, and established role in preclinical drug development. They possess well-documented metabolic enzyme profiles—particularly cytochrome P450 isoforms—that are functionally comparable to those in humans. Their consistent pharmacokinetic responses, genetic uniformity, and extensive historical data make them a reliable in vivo model for evaluating the absorption, distribution, metabolism, and excretion (ADME) of investigational compounds. Moreover, both FDA [11] and the International Council for Harmonisation (ICH) [12] recommend the use of male animals in early-stage nonclinical pharmacokinetic and toxicokinetic studies to minimize variability arising from the estrous cycle in females. This standardized approach enhances the consistency and interpretability of baseline pharmacokinetic data during the early phases of drug development.

Thus, in this study, we present a systematic, accurate mass spectrometry–based approach for metabolite identification that integrates both in vitro and in vivo methodologies. We evaluated the in vitro drug metabolism and Caco-2 cell permeability of R14, and investigated its in vivo metabolic pathways using male Sprague Dawley (SD) rats as the animal model. Comprehensive assessments of oral bioavailability, pharmacokinetic profiles, and metabolic transformations were conducted. This integrated strategy enabled a detailed characterization of R14’s disposition and provided critical insights to support early-stage drug development.

## 2. Materials and Methods

### 2.1. Chemicals and Reagents

R14 (CAS#: 727664-79-1) was purchased from EMD Chemicals, Inc. (San Diego, CA, USA). Citric acid, sodium sulfite, LC-MS grade solvents (acetonitrile, methanol, and water) were purchased from VWR Chemicals (Chicago, IL, USA). LC-MS grade formic acid was bought from Fisher Scientific (Pittsburgh, PA, USA). Phosphate-buffered saline (PBS), Hank’s Balanced Salt Solution (HBSS), NADPH, MgCl_2_, HBSS, glucose, HEPES and InVitroGRO™ CP Medium were obtained from Sigma-Aldrich (St. Louis, MO, USA). Blank rat plasma, rat blood, and rat liver microsomes were bought from BioIVT (Westbury, NY, USA). Primary human hepatocytes (male) were purchased from Thermofisher Scientific (Waltham, MA, USA). The NADPH regenerating system, DMEM medium, recombinant human CYP isoforms (CYP3A4, CYP3A5, CYP2C19, CYP1A2, CYP2B6), and 6-well cell insert plates were purchased from Corning (Glendale, AZ, USA). Cloned Caco-2 cells (ATCC-derived) were kindly gifted by Dr. Ming Hu (College of Pharmacy, University of Houston, Houston TX, USA). All other chemicals were of analytical reagent grade and sourced from commercial suppliers.

### 2.2. In Vitro Studies

#### 2.2.1. R14 Incubation in Rat Whole Blood

Previously, we observed the complete disappearance of R14 from mouse whole blood samples after being stored for 10 days in a −70 °C freezer, raising concerns about its stability under typical storage conditions. To further investigate the degradation and potential metabolic transformation of R14 in whole blood, we spiked rat whole blood with R14 (10 µg/mL) and left it at room temperature for 3 days. Room temperature incubation allows for ongoing enzymatic and non-enzymatic processes, providing insight into R14’s metabolic stability and degradation pathways under physiologically relevant but uncontrolled conditions. After incubation, a 4X volume of methanol containing 0.5% formic acid was added to the samples, followed by vortexing for 3 min and centrifugation to obtain supernatant prior to metabolite identification using the UHPLC coupled with quadrupole time-of-flight tandem mass spectrometry (UHPLC-QTOF MS/MS).

#### 2.2.2. Phase I Metabolism in Rat Liver Microsomes

The stock solution of R14 (5 µL, 1 mM) were pre-incubated with rat liver microsomes (2.5 µL, 20 mg/mL) and PBS (462.5 µL, 50 mM) for 5 min. Metabolism was then initiated by adding a NADPH regenerating system (25 µL of solution A and 5 µL of solution B). Samples were collected at various time points over 6 h. Aliquots (200 µL) were quenched with 100 µL of acetonitrile containing 0.6% formic acid for metabolite identification using UHPLC-QTOF MS/MS.

#### 2.2.3. CYP Isoform Identification

Five CYP isoforms, namely CYP1A2, 2B6, 3A4, 3A5, 2C19, were tested in this study. R14 (10 µL, 1 mM) was pre-incubated with each isoform (5 µL, 1 nmol/mL) and PBS (925 µL, 50 mM) for 5 min. Metabolism was initiated by adding a NADPH regenerating system (50 µL of solution A and 10 µL of solution B). Samples were collected at 0, 20, 40, 60, 120, 240, and 360 min. Aliquots (100 µL) were quenched with 50 µL of acetonitrile containing 0.6% formic acid and internal standard (IS), then analyzed by UPLC.

#### 2.2.4. Metabolic Kinetic Studies

To determine the metabolic kinetics, R14 was incubated with two major isoforms CYP3A4 and 2C19 at different concentrations in the above NADPH regenerating system. The half-life of parent R14 drug disappearance was calculated. Key parameters, including the Michaelis-Menten constant (K_m_), maximum reaction velocity (V_max_), and intrinsic clearance (Cl_int_) were obtained using the Michaelis–Menten model.

#### 2.2.5. R14 Metabolism in Human Hepatocytes

Twenty-four-well plates were precoated with rat tail collagen. Human hepatocytes suspended in InVitroGRO™ CP Medium were seeded into the plates at 0.2 × 10^6^ cells/well. Cells were grown in a CO_2_ incubator for 3 days, with the medium replaced every other day. After 3 days, the medium was removed, and the cells were washed 3 times with HBSS. The cells were then incubated with HBSS containing R14 at various concentrations (*n* = 3) at 37 °C. At 20 min, 40 min, 1 h, 2 h, 4 h, and 6 h, 100 µL aliquots were collected and quenched with 50 µL of acetonitrile containing 0.6% formic acid and IS. The samples were analyzed by UPLC to quantify R14 and its in vitro major metabolite (M27 and M46).

#### 2.2.6. R14 Permeability in the Caco-2 Cell Culture Model

The permeability of R14 was assessed using the Caco-2 cell monolayer model, following the protocol by Liu and Hu [13] (Liu 2002). Caco-2 cells (2.5 × 10^5^ cells per insert; 4.2 cm^2^ area, 3 μm pore size) were seeded and cultured at 37 °C with 90% humidity and 5% CO_2_ for 19–22 days. Monolayer integrity was evaluated using transepithelial electrical resistance (TEER) measurements with a Millicell ERS-2 system. Only monolayers with TEER values between 500 and 750 Ω·cm^2^ were used. Rutin (a low permeable compound) was used as an internal control to demonstrate the integrity of the monolayer.

Hank’s Balanced Salt Solution (HBSS), supplemented with NaHCO_3_ (0.35 g/L), HEPES (5.96 g/L), and glucose (3.5 g/L), adjusted to the desired pH, was used as the transport medium. Cells from passages 41–49 were used.

After confirming TEER, R14 working solution (10 µM) was added to either the apical or basolateral side of the insert, designating that side as the donor compartment, while the opposite side served as the receiver compartment. Samples (200 µL) were collected from both the apical (A) and basolateral (B) sides at 0, 0.5, 1, 2, and 4 h. The removed volume was immediately replaced with either fresh R14 working solution (on the donor side) or fresh buffer (on the receiver side) to maintain consistent volume and drug concentration throughout the experiment.

For analysis, 100 µL of acetonitrile containing 0.6% formic acid and internal standard was added to each collected sample. After vortexing and centrifugation, the supernatant was analyzed using UHPLC-MS/MS for R14 quantification.

The apparent permeability coefficient (*P*, cm/s) was calculated using the following equation:

$$P=\frac{dQ/dt}{{A\cdot C}_{0}}$$

where *dQ*/*dt* is the permeability rate of drug appearance in the receiver compartment (µmol/s), *A* is the surface area of the cell monolayer (cm^2^), and *C*_0_ is the initial concentration in the donor compartment (µmol/mL).

Permeability was assessed in both directions (apical to basolateral and basolateral to apical), and the efflux ratio was calculated as:

$$Effluxratio=\frac{P_{b-a}}{P_{a-b}}$$

where *P_a_*_−*b*_ is the permeability coefficient from apical to basolateral, and *P_b_*_−*a*_ is the permeability coefficient from basolateral to apical.

### 2.3. Pharmacokinetic Studies of R14 in Rats

#### 2.3.1. Animals

This study was carried out in accordance with the recommendation of the Guidelines for the Care and Use of Laboratory Animals (2011) and was in compliance with the ARRIVE guidelines. All animal experiments were conducted with prior approval from the Texas Southern University Institutional Animal Care and Use Committee (IACUC). Male adult SD rats (250–350 g) were purchased from Envigo RMS (Indianapolis, IN, USA) and were housed in standard rodent cages (3–4 rats per cage) with a 12 h light/dark cycle at 22–24 °C and had free access to standard laboratory rodent chow (7012 Teklad mouse/rat diet from Envego, Inc., Indianapolis, IN, USA) and water. Rats were acclimated to the animal care facility for at least 7 days before the start of each experiment. Only male rats were used in the present study to minimize pharmacokinetic variations due to sex differences.

#### 2.3.2. Surgical Procedures

To facilitate the timed withdrawal of multiple blood samples from each animal, the right jugular vein of each animal was cannulated one day prior to drug administration. The cannulas were flushed daily with 0.5 mL of sterile heparinized saline (100 units/mL). Under anesthesia using a cocktail of ketamine/acetopromazine/xylazine (50:3.3:3.3 mg/kg ip), silicone elastomer tubing (0.02 × 0.037 in) was inserted into the jugular vein, secured with a silk suture, and exteriorized in the dorsal intrascapular area. The surgical incision was prophylactically treated with nitrofurazone wound powder and closed with surgical staples.

#### 2.3.3. Dosing and Sampling

For the oral pharmacokinetics of R14, rats were fasted overnight and 4 h post-administration of 50 mg/kg oral dose of R14, with free access to water in metabolic cages. Serial blood samples (0.15 mL each) were collected from the cannulated jugular vein into heparin-coating tubes before dosing and at 5 min, 15 min, 30 min, 45 min, 1 h, 2 h, 3 h, 4 h, 6 h, 8 h, 10 h, and 24 h time points post dose. Plasma samples were separated immediately by centrifugation of the blood samples at 13,000 rpm for 3 min. For R14 quantification, 10 μL plasma aliquots were mixed with 30 μL of a stabilizing derivatization solution (0.1 M citric acid and 0.1 M sodium sulfite) and stored at −80 °C for analysis by UHPLC-MS/MS. For metabolite identification, 20 μL plasma aliquots were mixed with 80 μL acetonitrile containing 0.5% formic acid and stored at −80 °C for UHPLC-QTOF MS/MS analysis. Urine samples were collected over 0–24 h and stored at −80 °C.

For the intravenous (i.v.) pharmacokinetics of R14, another group of jugular vein cannulated rats received an i.v. administered 5 mg/kg R14 dose. Serial blood samples (0.15 mL each) were collected from the cannulated jugular vein into heparin-coating tubes before dosing and at 2 min, 5 min, 10 min, 30 min, 45 min, 1 h, 2 h, 4 h, 6 h, 8 h, 10 h, and 24 h time points post-dose. Plasma was processed similarly, with aliquots prepared for both R14 quantification and metabolite analysis, and urine collected over 24 h and stored at −80 °C.

### 2.4. R14 Quantification Using UHPLC-MS/MS

Due to the known instability of R14 in biological matrices, we previously developed and fully validated a stability-indicating LC-MS/MS method for its quantification in mouse whole blood, as described in our earlier publication [6]. In the present study, a partial validation was conducted to accommodate the change in matrix (rat plasma), in alignment with FDA and EMA bioanalytical method validation guidelines. Key parameters assessed included selectivity, calibration curve linearity, intra-day precision and accuracy at four quality control (QC) levels (LLOQ LQC, MQC, and HQC), as well as extraction recovery and matrix effects. The results confirmed acceptable performance of the method within regulatory criteria, thereby supporting its suitability for quantitative analysis of R14 in the rat plasma. Detailed validation data are provided in the Supplemental Information.

#### 2.4.1. Preparation of Stock Solutions

Stock solutions of the analyte (1 mg/mL) and IS (warfarin, 500 μg/mL) were prepared in acetonitrile, respectively, and stored at −20 °C until used. Standard samples were prepared by diluting the R14 stock solution with acetonitrile and spiking into blank rat plasma to achieve concentrations of 1, 5, 10, 50, 100, 500 and 1000 ng/mL. The IS working solution was prepared by diluting warfarin stock solution with acetonitrile to obtain a concentration of 2.5 ng/mL.

#### 2.4.2. Sample Preparation

The urine sample (10 μL each) was pre-mixed with 30 µL of derivatization agent (also acting as stabilizing agent). Pre-mixed plasma samples (with the derivation agent before storage) or the pre-mixed urine samples were added to 360 µL of acetonitrile (containing 2.5 ng/mL warfarin), vortexed for 2 min, and then centrifuged for 20 min. The supernatant (80 μL) was mixed with 160 µL of acetonitrile containing 0.5% formic acid, and finally, 100 μL aliquot of the mixture was transferred to a sample vial for UHPLC-MS/MS analysis.

#### 2.4.3. UHPLC-MS/MS Conditions

R14 quantification via monitoring R14 bisulfite was conducted using a previously published method on a 6500+ Triple Quad UHPLC-MS/MS System (SCIEX, Framingham, MA, USA) coupled with a Synergi Fusion-RP column (50 × 2 mm, 4 µm, 80 Å, Phenomenex Inc., Torrance, CA, USA) [6].

### 2.5. Quantification of R14 and Metabolites Using UPLC

Quantification of R14 and its metabolites in human hepatocytes and CYP isoforms was performed on an Acquity UPLC system (Waters Co., Milford, MA, USA) with a C18 column (2.1 × 50 mm, Acquity BEH C18, 1.7 µm, Waters Co., Milford, MA, USA). The mobile phases were: A—water with 0.1% formic acid (*v*/*v*), and B—acetonitrile with 0.1% formic acid (*v*/*v*). The gradient program started at 10% B for 1 min, ramped to 90% in 3 min, kept at 90% for 0.3 min, returned to 10% in 0.2 min, and equilibrated for 0.5 min. The Flow rate was 0.45 mL/min. The autosampler and column temperatures were maintained at 10 °C and 40 °C, respectively. Formononetin was used as the IS. The linear quantification range for R14 was 0.078–10 µM. Concentrations of M27 and M46 were estimated using R14 calibration curve, adjusted by conversion factors based on their respective extinction coefficients [14].

### 2.6. Identification of Unknown Metabolites of R14 Using UHPLC-QTOF MS/MS

#### 2.6.1. Urine Sample Preparation

Twenty microliters of urine were mixed with 80 µL of methanol containing 0.5% formic acid, vortexed for 2 min, and centrifuged for 20 min. An 80 µL Aliquot of the supernatant was transferred to a sample vial for metabolite identification using UHPLC-QTOF MS/MS.

#### 2.6.2. Plasma, Blood, and Microsomal Sample Preparation

Samples from in vivo pharmacokinetic studies, in vitro blood stability studies, and in vitro hepatic microsome studies were pretreated at the time of collection. Before analysis, the samples were thawed at room temperature, vortexed for 2 min, and centrifuged at 14,000 rpm for 20 min. An 80 µL aliquot of the supernatant was transferred to a sample vial for UHPLC-QTOF MS/MS analysis.

#### 2.6.3. Instrumentation and Analytical Conditions

Metabolite identification was performed on a UHPLC-X500B QTOF MS/MS (SCIEX, Framingham, MA, USA) equipped with a Synergi Fusion-RP column (4 µm, 150 × 2 mm, 80 Å, Phenomenex, Torrance, CA, USA). The mobile phases were water with 0.1% formic acid (A) and methanol with 0.1% formic acid (B), delivered at a flow rate of 0.4 mL/min. The autosampler and oven temperatures were set at 10 °C and 30 °C, respectively. The running time was 30 min. The gradient elution was as follows: 10% B for 0.5 min; increased to 70% over 20 min; then to 95% over 2 min; held at 95% for 5 min; returned to 10% in 0.5 min; and equilibrated for 2 min.

The QTOF MS/MS was operated In positive ion mode. Data were acquired in Information-dependent-acquisition (IDA) mode, starting with a TOF MS survey scan to detect precursor ions, followed by MS/MS scans of the top 10 most intense ions (intensity > 200 counts/s). TOF MS was set to scan *m*/*z* 200–700 with a 0.1 s accumulation time, while TOF MS/MS scanned *m*/*z* 50–700 with a 0.04 s accumulation time. The source temperature and spray voltage were set at 500 °C, and 5000 v, respectively. Curtain gas and collision gas (CAD) were set at 30 and 9 psi, while nebulizer gas (gas 1) and heater gas (gas 2) were set at 55 and 60 psi, respectively. The declustering potential (DP) was 50 V. Collision energy (CE) were 10 v for TOF MS and 35 v (±15 v spread) for TOF MS/MS. Data were acquired using SCIEX OS software 1.6.1, and processed on SCIEX OS software 3.1.0.

#### 2.6.4. Strategies for R14 Unknown Metabolites’ Identification

**Figure 1:**
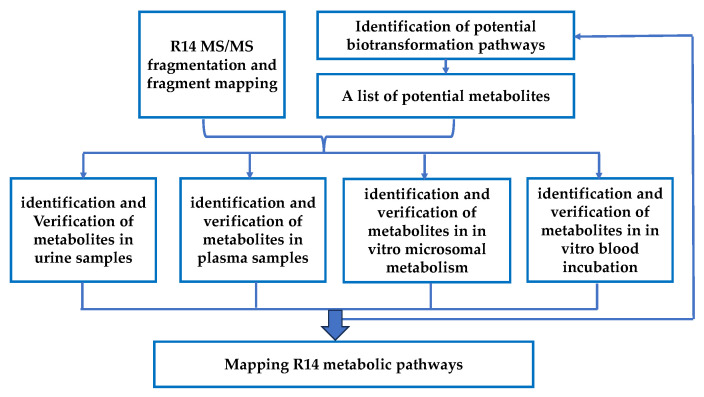
Workflow for metabolite identifications and metabolic pathway mapping.

To elucidate the metabolic pathways and identify unknown metabolites of R14, we employed a multi-step strategy. First, MS/MS fragmentation analysis was performed to characterize the fragmentation pattern of R14 and identify its key fragment ions, which served as references for metabolite identification. Next, based on the chemical structure of R14, potential biotransformation pathways mediated by hepatic cytochrome P450 enzymes were predicted. These predicted metabolites were then screened in urine samples collected during in vivo pharmacokinetic studies to identify excreted metabolites. Metabolites detected in urine were subsequently cross-verified in plasma samples from the same in vivo studies. If verification failed or additional metabolites were suspected, the analysis reverted to the initial fragmentation step to identify downstream or daughter metabolites. To confirm the metabolic origin and biological relevance of in vivo–identified metabolites, cross-validation was performed using in vitro hepatic microsomal and blood incubation studies. Finally, all confirmed metabolites were compiled into a comprehensive metabolic pathway map of R14 (Figure 1).

### 2.7. Pharmacokinetic Analysis

Noncompartmental pharmacokinetic parameters [biological half-life (T_½_), plasma clearance (Cl), volume of distribution (V_d_), total area under the plasma concentration–time curve (AUC), and mean residence time (MRT)] were determined using Phoenix WinNonlin v8.0 software (Pharsight Corporation, Mountain View, CA, USA). The maximum plasma concentration (C_max_) and the time to reach maximum plasma drug concentration (T_max_) were determined from the plasma drug concentration versus time profile. Oral bioavailability F = (AUC_oral_∙Dose_i.v._)/(AUC_i.v._∙Dose_oral_)]. Cl/F was determined as Dose/AUC, where Cl = drug clearance and F = oral bioavailability of R14.

### 2.8. Use of Generative AI Tools

During the preparation of this manuscript, ChatGPT (GPT-4o, OpenAI, San Francisco, CA, USA) was occasionally used to improve the clarity, grammar, and language structure of the text. The authors reviewed and edited the AI-generated content to ensure accuracy and scientific integrity. No AI tools were used for study design, data generation, data analysis, or interpretation.

## 3. Results

### 3.1. Pharmacokinetics of R14

**Figure 2:**
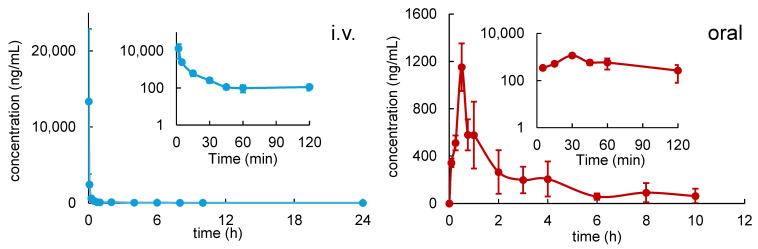
Mean concentration–time profiles of R14 in mouse whole blood after i.v. (5 mg/kg) and oral (50 mg/kg) administrations of R14 (*n* = 3).

Following i.v. bolus dose, R14 showed bi-exponential disposition with a rapid distribution phase followed by a slower elimination phase of a mean T_1/2_ 5.39 h in rats. Absorption of R14 after oral administration was fast and reached a mean C_max_ of 1152 ng/mL at 0.5 h post dose. Absolute oral bioavailability of R14 was estimated to be 13.4% in rats. Less than 0.05% of either i.v. or oral administered R14 doses were excreted in the 24 h urine samples (Figure 2 and Table 1).

### 3.2. Fragmentation of the Parent Drug R14

**Figure 3:**
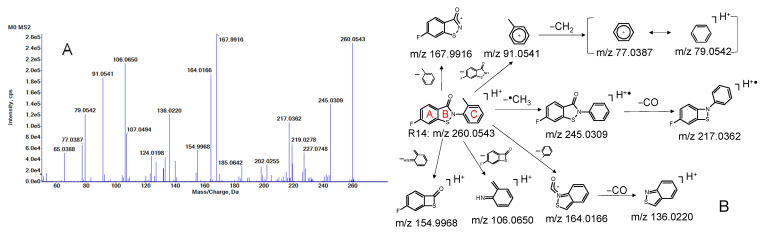
HR MS/MS of the parent R14 (**A**), and fragmentation pathways of R14 (**B**).

R14 is a tricyclic compound composed of a fluorine-substituted phenyl ring (Ring A) fused with a γ-lactam containing a sulfur atom (Ring B), and a para-methylphenyl ring (Ring C) attached to Ring B via the nitrogen atom (Figure 3B). HR MS and MS/MS spectra of R14 were acquired using UHPLC-QTOF MS/MS. R14 eluted at a retention time (RT) of 19.6 min, with a protonated molecular ion ([M + H]^+^) observed at *m*/*z* 260.0539, corresponding to the molecular formula C_14_H_10_FNOS (calculated [M + H]^+^ at *m*/*z* 260.0540; mass error: −0.3 ppm).

Prominent fragment ions were detected at *m/z* 260.0543, 245.0309, 217.0362, 167.9916, 164.0166, 154.9968, 136.0220, 106.0650, 91.0541, 79.0542, and 77.0387 (Figure 3A). The proposed fragmentation pathways are illustrated in Figure 3B. Briefly, the fragment at *m*/*z* 245.0309 (calculated *m*/*z* 245.0305) resulted from the loss of a methyl radical (–●CH_3_) from R14, followed by the loss of carbon monoxide (–CO) to yield the ion at *m*/*z* 217.0362 (calculated *m*/*z* 217.0356). Cleavage of the C–N bond linking Rings B and C generated ions at *m*/*z* 167.9916 (calculated *m/z* 167.9914) and 91.0541 (calculated *m*/*z* 91.0542), with subsequent fragmentation yielding ions at *m*/*z* 79.0542 and 77.0387. The ion at *m*/*z* 164.0166 (calculated *m*/*z* 164.0165) arose from the loss of a fluorophenyl group (–C_6_H_5_F), and further loss of CO produced the ion at *m*/*z* 136.0220 (calculated *m*/*z* 136.0216). Ions at *m*/*z* 154.9968 and 106.0650 were attributed to fragmentation at the S–N and C–N bonds, respectively.

The R14 MS/MS fragment patterns guided us in further structural elucidation for unknown metabolites.

### 3.3. Identification of R14 Metabolites

**Figure 4:**
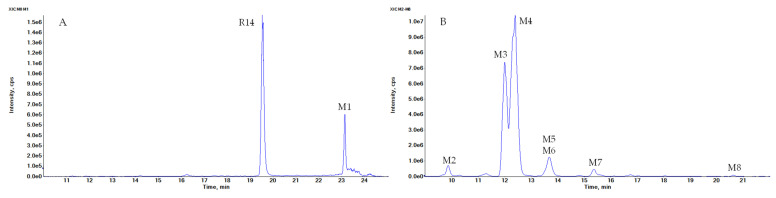

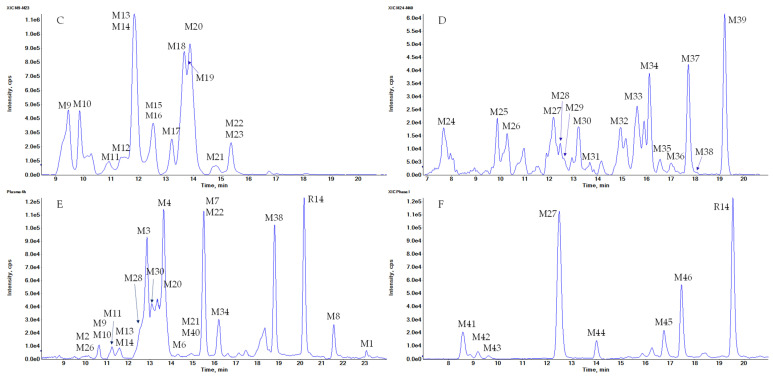
Extracted ion chromatograms of R14 metabolites. (**A**) After incubation of R14 with rat whole blood; (**B**) metabolites in a urine sample at the interval 0–24 h after a single oral dose of 50 mg/kg R14 (distinguishable peaks that Peak height up to 1.0 × 10^7^); (**C**) metabolites in a urine sample at the interval 0–24 h after a single oral dose of 50 mg/kg R14 (Peak height between 5.0 × 10^4^–1.0 × 10^6^); (**D**) metabolites in a urine sample at the interval 0–24 h after a single oral dose of 50 mg/kg R14 (Peak height lower 6.0 × 10^4^); (**E**) a plasma sample at 4 h after a single oral dose of 50 mg/kg R14; (**F**) an in vitro sample of phase I metabolism in rat liver microsomes.

Metabolite prediction on MS/MS fragmentation data of R14 was conducted using MetabolitePilot software (version 2.0.4, SCIEX). Analysis of MS and MS/MS chromatograms from rat whole blood incubations revealed a predominant metabolite, designated M1. Structural elucidation indicated that M1 is a hydrogenation product of R14, formed by the reduction of its carbonyl group (Figure 4A). Phase I metabolism studies using hepatic microsomes identified two additional major metabolites: M27 and M46, resulting from mono-hydroxylation of R14 on ring A and ring C, respectively. These findings suggest that R14 undergoes three primary metabolic transformations in vivo, yielding M1, M27, and M46 as key metabolites before further biotransformation. Each metabolic pathway was further characterized, and all predicted metabolites were confirmed through comprehensive MS analysis, including accurate mass and isotope pattern matching using the Formula Finder tool in SCIEX OS software, as well as MS/MS fragment ion matching via the Fragments Pane. The mass errors between observed and calculated values were all within 5 ppm, providing high confidence in the proposed elemental compositions.

The following characteristic fragmental ions provided key role in proposing the substitution position: calculated *m*/*z* 154.9961 ([FC_6_H_3_COS+H]^+^) for non-substitution on Ring A, calculated *m*/*z* 169.0118 ([CH_3_FC_6_H_2_COS+H]^+^) for mono-methylation on ring A, calculated *m*/*z* 185.0067 ([CH_3_(OH)FC_6_HCOS+H]^+^) for mono-methylation and mono-hydroxylation on Ring A, calculated *m*/*z* 201.0016 ([CH_3_(OH)_2_FC_6_COS+H]^+^) for mono-methylation and di-hydroxylation on Ring A, calculated *m*/*z* 106.0651 ([NC_6_H_4_CH_3_+H]^+^) for non-substitution on Ring C, calculated *m*/*z* 122.0600 ([NC_6_H_3_CH_3_OH+H]^+^) for mono-hydroxylation on Ring C, and calculated *m*/*z* 138.0550 ([NC_6_H_2_CH_3_(OH)_2_+H]^+^) for di-hydroxylation on Ring C. The neutral losses of 79.9563 Da (SO_3_) and 176.0315 Da (C_6_H_8_O_6_) were used for the characterization of sulfation and glucuronidation, respectively.

**Figure 5:**
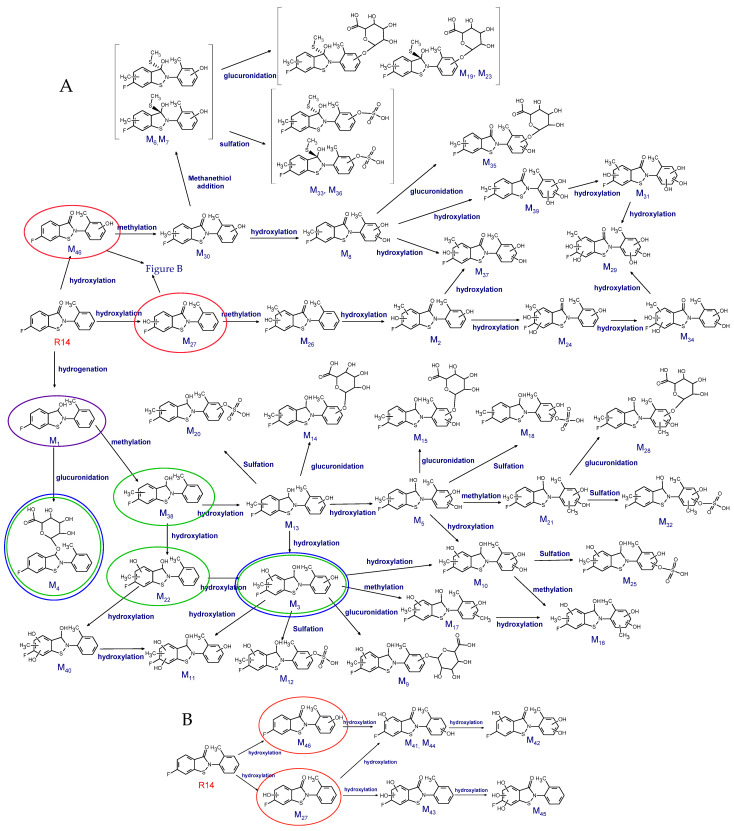
Proposed metabolic pathways of R14. (**A**) Comprehensive metabolic map of R14 biotransformation. (**B**) Proposed Phase I metabolic pathways of R14. Major metabolites identified in various biological matrices are highlighted using color-coded circles: green, major metabolites in rat plasma; blue, major metabolites in rat urine; purple, predominant metabolite following R14 incubation in rat whole blood; red, major metabolites identified in Phase I in vitro metabolic reaction samples.

R14 predominantly underwent multiple Phase I hydroxylation and Phase II methylation reactions, followed by further Phase II conjugation through glucuronidation and sulfation. A total of 46 metabolites were identified. Among these, M1 was the dominant metabolite observed following R14 incubation in rat whole blood (Figure 4A). In urine samples, 38 metabolites were detected (Figure 4B–D), while 21 metabolites were identified in plasma (Figure 4E). Additionally, eight metabolites were detected in an in vitro Phase I metabolic reaction (Figure 4F). The elemental compositions, observed and calculated exact masses, mass errors, and characteristic fragment ions for the proposed metabolites are summarized in Table 2. The proposed metabolic pathways of R14 are illustrated in Figure 5.

Detailed procedures for individual metabolite identification and confirmation are provided in the Supplementary Information. Four representative metabolites are highlighted below.

**Figure 6:**
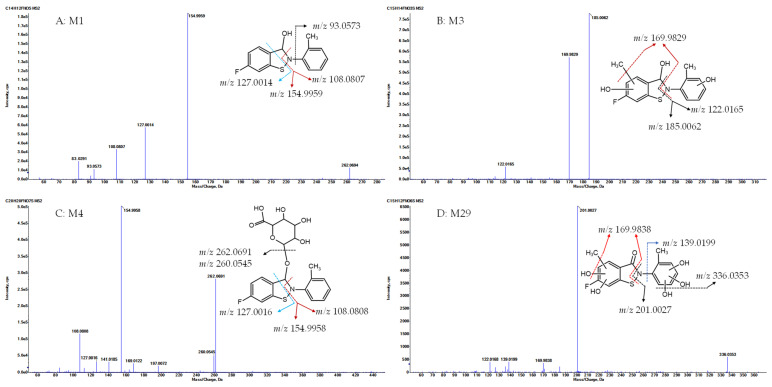
HR MS/MS of typical metabolites and fragmentation pathways.

- Metabolite M1, with a protonated ion at *m*/*z* 262.0695, was eluted at 23.1 min in the UHPLC-QTOF MS/MS analysis and was identified as the sole major metabolic product following the incubation of R14 in blank rat whole blood for 3 days at room temperature (Figure 4A). In contrast, M1 was detected at relatively low levels in the in vivo plasma samples. The proposed chemical formula for M1 is C_14_H_12_FNOS, corresponding to a calculated [M + H]^+^ *m*/*z* of 262.0696, with a mass error of −0.5 ppm. M1 was identified as a hydrogenation product of R14, resulting from the reduction of the carbonyl functional group. MS/MS fragmentation supported this structural assignment, showing characteristic fragment ions at *m*/*z* 154.9959, 127.0014 ([FC_6_H_3_S+H]^+^, calculated *m*/*z* 127.0012), and 108.0807 ([C_6_H_4_CH_3_NH_2_+H]^+^, calculated *m*/*z* 108.0808) (Figure 6A).

- Metabolite M3, with a protonated ion at *m*/*z* 308.0750, was identified at a retention time of 12 min in the urine samples analyzed by UHPLC-QTOF MS/MS. The proposed molecular formula for M3 is C_15_H_14_FNO_3_S, corresponding to a calculated [M + H]^+^ *m*/*z* of 308.0751, with a mass error of –0.4 ppm. This composition suggests that M3 is a mono-methylated and di-hydroxylated derivative of M1. In the MS/MS spectrum, key fragment ions were observed at *m*/*z* 185.0062 and 169.9829, indicating that mono-methylation and mono-hydroxylation occurred on ring A, while the additional hydroxylation likely took place on ring C (Figure 6B).
- Metabolite M4, with a protonated ion at *m*/*z* 438.1017, was eluted at 12.3 min in the UHPLC-QTOF MS/MS analysis of rat urine samples. The proposed molecular formula for M4 is C_20_H_20_FNO_7_S, corresponding to a calculated [M + H]^+^ *m*/*z* of 438.1017, with a mass error of –0.1 ppm. The presence of an additional C_6_H_8_O_6_ moiety (176.0315 Da) compared to M1 suggests that M4 is a glucuronide conjugate of M1. MS/MS fragmentation further supported this structure, with characteristic ions observed at *m*/*z* 262.0691 ([M1 + H]^+^), 154.9958, 127.0016, and 108.0808, confirming M4 as the glucuronide conjugate of M1 (Figure 6C).
- Metabolite M29, with a protonated ion at *m*/*z* 354.0442, was eluted at 12.7 min in the UHPLC-QTOF MS/MS analysis of rat urine samples. The proposed molecular formula for M29 is C_15_H_12_FNO_6_S, corresponding to a calculated [M + H]^+^ *m*/*z* of 354.0442, with a mass error within acceptable limits. This composition indicates the addition of a CH_2_O_5_ moiety relative to R14, suggesting that M29 may be a mono-methylated and penta-hydroxylated derivative of R14, or alternatively, a mono-hydroxylation product of either M31 or M34. MS/MS fragmentation of M29 revealed key product ions at *m*/*z* 201.0027, 336.0353, and 169.9838. These fragments support the presence of a mono-methyl group and two hydroxyl substitutions on ring A, with the remaining three hydroxyl groups located on ring C. Based on this fragmentation pattern, M29 is proposed to be a mono-hydroxylation product of M31 (on ring A) or M34 (on ring C) (Figure 6D).

### 3.4. In Vitro Metabolism of the Two Major Phase I Metabolites, M27 and M46

#### 3.4.1. Metabolism of M27 and M46 in Human Hepatocytes

**Figure 7:**
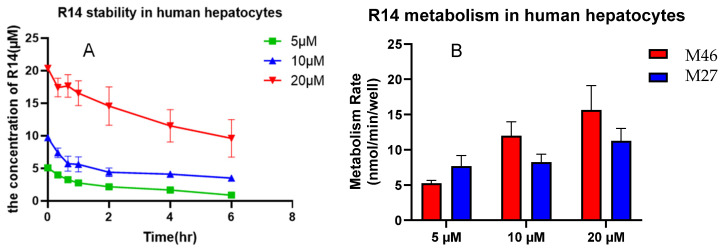
R14 metabolism with human hepatocytes. R14 stability in human hepatocytes (**A**), formation rate of M27 and M46 (**B**).

Metabolites M27 and M46 were observed at relatively high abundance during Phase I metabolism in rat liver microsomes (Figure 4F). When R14 was incubated with human hepatocytes, both M27 and M46 were also detected as major metabolites, suggesting that they may represent primary metabolic products in the human liver. Furthermore, the metabolic profiles of these metabolites remained consistent and proportional across R14 concentrations ranging from 5 to 20 µM (Figure 7), indicating concentration-independent formation within this range.

#### 3.4.2. CYP Isoforms Involvement in M27 and M46 Formation

Five CYP isoforms—CYP1A2, 2B6, 3A4, 3A5, and 2C19—were tested in this study based on their clinical relevance and substantial roles in human hepatic drug metabolism. These isoforms collectively account for a significant portion of phase I metabolism, particularly in the oxidative biotransformation of xenobiotics. CYP3A4 and CYP3A5 are among the most abundant in the liver and intestine; CYP1A2 and CYP2B6 are involved in the metabolism of numerous drugs and environmental chemicals; and CYP2C19 plays a key role in metabolizing several clinically important drugs. While other major isoforms such as CYP2C9 and CYP2D6 were considered, they were excluded in this initial study to prioritize isoforms with the highest relevance to the expected metabolic pathways of R14, as suggested by preliminary in silico predictions and structural analog comparisons.

**Figure 8:**
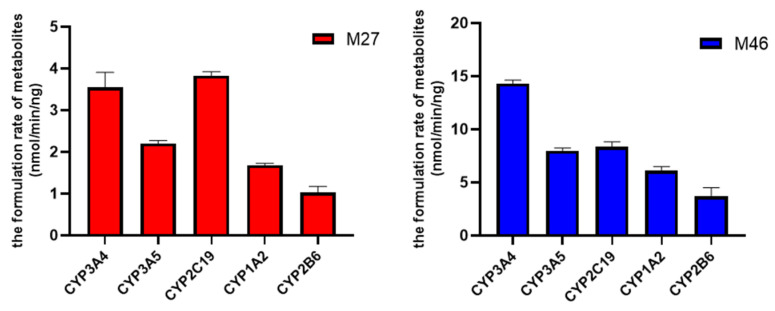
Formation rates of M27 and M46 when R14 (10 µM) were incubated with different recombinant human CYP isoforms (*n* = 3).

When incubated R14 with the five major CYP isoforms, M27 and M46 were afforded, suggesting that these two major metabolites were generated by CYPs. The results also showed that for M27, the formation rates were about 2-folds higher when incubated with CYP3A4 and 2C19 than incubated with the other three isoforms. Similarly, the formation rate of M46 was highest when incubating R14 with CYP3A4. These findings suggested that CYP3A4 and 2C19 were two major isoforms to generate M27 and M46 (Figure 8). Incubation of R14 with the five major CYP isoforms resulted in the formation of both M27 and M46, indicating that these metabolites are CYP-mediated products. The formation rate of M27 was approximately two-fold higher when incubated with CYP3A4 and CYP2C19 compared to the other three isoforms. Similarly, M46 formation was most prominent in the CYP3A4 incubation. These results suggest that CYP3A4 and CYP2C19 are the primary isoforms responsible for the biotransformation of R14 into M27 and M46 (Figure 8).

#### 3.4.3. Enzyme Kinetics of M27 and M46 Formation

**Figure 9:**
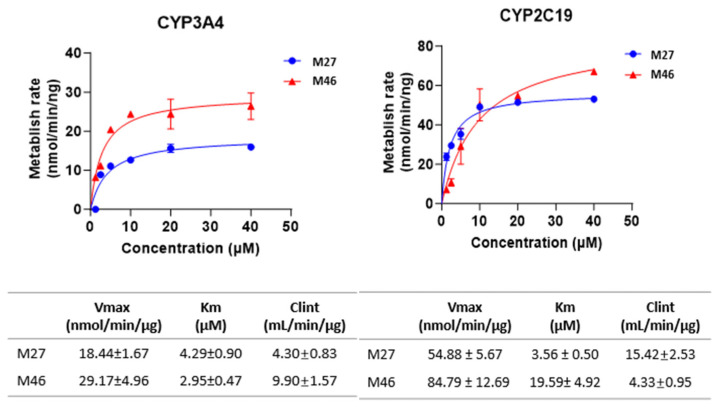
Michaelis–Menten kinetic parameters (K_m_, V_max_, and Cl_int_) of R14 metabolism in CYP3A4 and CYP2C19 reaction systems. Data was obtained from nonlinear regression analysis of metabolite formation rates across varying substrate concentrations.

Kinetic studies using multiple substrate concentrations revealed that the mean Cl_int_ values for M27 and M46 formation with CYP3A4 were 4.3 and 9.9 mL/min/µg, respectively. In comparison, the Cl_int_ values for the same metabolites with CYP2C19 were 15.4 and 4.3 mL/min/µg, respectively (Figure 9). These data further support the involvement of CYP3A4 and CYP2C19 as key enzymes in the Phase I metabolism of R14.

### 3.5. R14 Permeability in the Caco-2 Cell Culture Model

**Figure 10:**
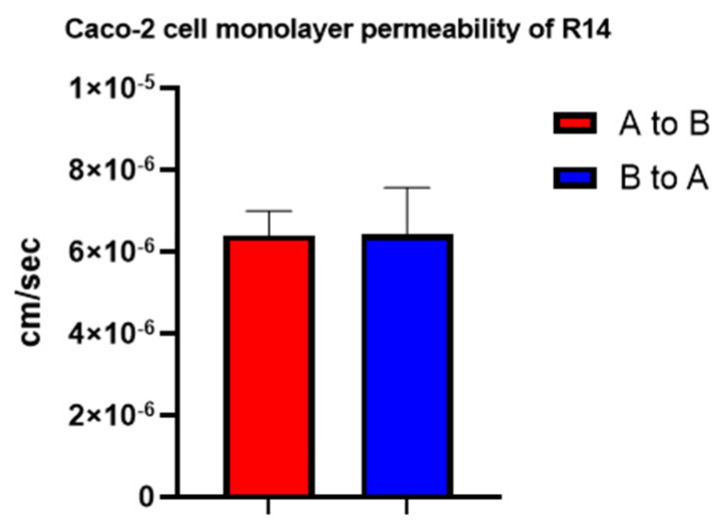
R14 permeability in the Caco-2 cell culture model (*n* = 3).

The permeability of R14 was evaluated using the Caco-2 cell monolayer model, a well-established in vitro system for predicting intestinal drug absorption in humans. At a concentration of 10 µM, R14 exhibited an apical-to-basolateral permeability (*P_a_*_−*b*_, the uptake permeability) of 6.39 ± 0.60 × 10^−6^ cm/s and a basolateral-to-apical permeability (*P_b_*_−*a*_, the efflux permeability) of 6.41 ± 1.15 × 10^−6^ cm/s. The calculated efflux ratio (*P_b_*_−*a*_/*P_a_*_−*b*_) was 1.12, indicating that active efflux transporters were unlikely to significantly affect R14 transport across the intestinal epithelium. These results suggest that R14 exhibits moderate permeability, and limited intestinal absorption is unlikely to be the major contributor to its low oral bioavailability (Figure 10).

## 4. Discussion

R14 was rapidly and extensively metabolized in vivo. Following i.v. administration, the mean systemic clearance (CL) and volume of distribution (Vd) of R14 were 2.63 L/h/kg and 20.70 L/kg, respectively. Notably, the systemic clearance exceeded the average hepatic blood flow in rats (2.55 L/h/kg) [15], indicating that R14 is a high-extraction ratio drug. In contrast, after oral administration, the apparent clearance (CL/F) and apparent volume of distribution (V/F) were markedly higher (22.04 L/h/kg and 93.35 L/kg, respectively) than those observed after intravenous dosing. These differences are attributed to the low observed oral bioavailability (13.4%). As a result, CL/F and V/F are overestimated relative to their true systemic values. This phenomenon is commonly observed when absorption is incomplete, variable, or subject to significant first-pass metabolism [16,17]. Moreover, variability in gastrointestinal transit, metabolism in the gut wall or liver, and efflux transport can all contribute to lower systemic exposure and apparent alterations in pharmacokinetic parameters after oral dosing [18]. Additionally, the small sample size (*n* = 3) represents a limitation of this study and may have contributed to variability in the pharmacokinetic estimates. Further studies with larger cohorts will be conducted to confirm these findings and to better characterize the pharmacokinetic profile of R14.

To further investigate the cause of low oral bioavailability, in vitro studies using the Caco-2 cell culture permeation model predicted moderate gastrointestinal absorption of R14, suggesting that limited membrane permeability is unlikely to be the primary barrier. Multiple plasma concentration peaks were detected at approximately 4 and 8 h after oral administration. Enterohepatic recirculation was ruled out, as R14 lacks the functional groups typically associated with this process. One possible explanation for the multiple peaks is the dissolution behavior of R14 in the gastrointestinal tract, potentially due to its low water solubility, or to continued absorption in the lower gastrointestinal regions.

Furthermore, intestinal metabolism may also contribute to the low oral bioavailability. Although hepatic clearance appears to be the dominant route of elimination, the potential role of drug-metabolizing enzymes present in enterocytes—such as intestinal cytochrome P450 isoforms—cannot be excluded. To further elucidate the contribution of intestinal first-pass metabolism, additional studies using intestinal microsomes or in situ intestinal perfusion models are planned.

Male rats were used for this initial pharmacokinetic and metabolism studies in accordance with FDA and ICH guidance. Given R14’s intended use for breast cancer treatment, pharmacokinetic studies in female animals are planned to assess sex-based differences and provide a more complete preclinical evaluation.

When studying a new drug candidate like R14, it is crucial to understand the significance of its major metabolic pathways—specifically, the amount or fraction of major metabolites and their dynamic behavior in vivo. This information not only helps in identifying potential toxicity profiles of R14 but also in assessing its efficacy, particularly if any metabolites are pharmacologically active.

While a mass balance pharmacokinetic study would indeed provide a comprehensive assessment of the in vivo distribution of the metabolites, it was not feasible at this early, exploratory stage of the investigation. In its place, we employed a relative estimation strategy using UHPLC-QTOF MS peak areas to evaluate trends in metabolite formation in plasma and urinary excretion. Due to the absence of authentic standards, we acknowledge that differences in ionization efficiency between the parent compound and its metabolites, as well as matrix effects—including potential signal suppression or enhancement from co-eluting components—may impact quantitative accuracy. Despite these limitations, this semi-quantitative approach is commonly employed in early-phase drug development for providing a preliminary understanding of systemic exposure and identifying major circulating or excreted metabolites for prioritization in future targeted studies.

**Figure 11:**
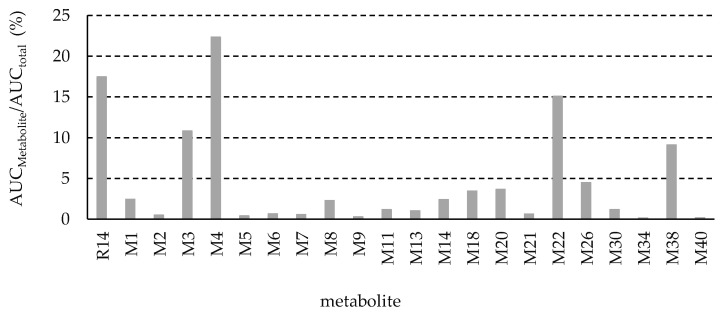
Apparent metabolites’ exposure in plasma compared with total drug-related exposure. Data was generated using peak area of extracted ions in UHPLC-QTOF MS analysis as relative concentration.

The UHPLC-QTOF MS peak area versus time data for the 21 metabolites identified in rat plasma were used to estimate the apparent AUC of each metabolite using WinNonlin software, with peak area serving as a proxy for relative concentration. Figure 11 presents a percentage comparison of the AUC of each identified metabolite relative to the total AUC of R14 and all its identified metabolites. Four metabolites—M3 (10.8%), M4 (22.3%), M22 (15.1%), and M38 (9.1%)—were identified as major plasma metabolites. All other metabolites were present at levels below 5% of total drug-related exposure. According to regulatory guidance, a metabolite is generally considered major if its AUC exceeds 10% of the total drug-related exposure [10]. Although M38 accounted for only 9.1%—slightly below this threshold—it was still classified as a major metabolite to minimize potential inaccuracies in estimation.

**Figure 12:**
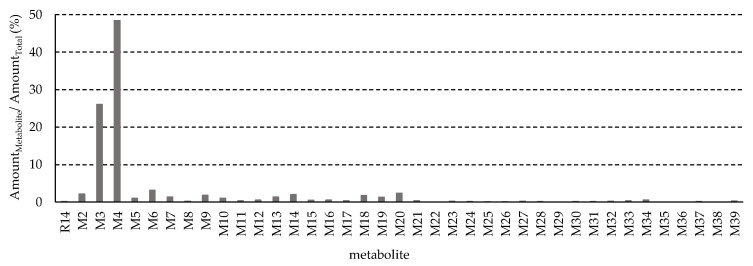
Cumulative fraction of excreted metabolites in urine (amount was estimated using peak area multiple collected urine volume during 24 h).

Figure 12 shows the cumulative excretion of metabolites in 24 h urine samples, expressed as a fraction of the total amount of R14 and its metabolites. The cumulative amount was estimated by multiplying the UHPLC-QTOF MS peak area by the total urine volume collected over the 24 h period. R14 was extensively metabolized, with less than 1% of the unchanged parent drug detected in the urine. All identified metabolites were excreted in greater amounts than R14 itself. M3, a methylation and hydroxylation product of R14, and M4, a glucuronide conjugate of the R14 hydrogenation product M1, were identified as major urinary metabolites. Their estimated relative contributions to the total urinary excretion were 26.1% for M3 and 48.4% for M4, respectively.

The major metabolites of R14 identified from in vitro liver microsomal incubations were M27 and M46, which were consistent with in silico predictions using ADMET Predictor v12.0 software. This tool, which simulates metabolism in human liver microsomes, also predicted M27 and M46 as major metabolites, supporting our in vitro findings.

Another metabolite, M1, was identified as a reduction product of R14 via hydrogenation of the carbonyl group. It was a major product following incubation of R14 in blank rat whole blood, suggesting that its formation may result from a chemical rather than an enzymatic reaction. R14 is chemically unstable in the presence of reducing agents, a property previously utilized in a chemical derivatization method for its quantification, as reported in our published work. Despite the likelihood of a non-enzymatic origin, M1 was also detected in plasma samples and represents an important intermediate; therefore, we still consider it a metabolite in our analysis.

A significant discrepancy was observed between the in vitro and in vivo results. The three major in vitro metabolites likely represent intermediate compounds in the in vivo metabolic pathway. Specifically, our in vivo data showed that M1 was undetectable in urine and present at only low concentrations in plasma; M27 was undetectable in plasma and detected at low levels in urine; and M46 was absent from both urine and plasma. These differences suggest that metabolites such as M1, M27, and M46 are transient intermediates that undergo rapid and extensive downstream metabolism in vivo—metabolic processes that are not fully recapitulated under in vitro conditions.

Drug metabolism in vivo is inherently complex and often involves multi-step biotransformations. For R14, three principal Phase I pathways were identified: hydroxylation of the aromatic rings and reduction of the carbonyl group, leading to the formation of M27 and M46 (via CYP450-mediated hydroxylation) and M1 (via carbonyl reduction), respectively. These intermediates were further processed through sequential methylation reactions—a Phase II transformation—interspersed with additional hydroxylation. Ultimately, the metabolites underwent terminal conjugation via glucuronidation and sulfation, completing the metabolic pathway.

The observed discrepancies between in vitro and in vivo profiles may arise from factors such as differential enzyme expression, cofactor availability, or extrahepatic metabolism in tissues such as the kidney, intestine, or lung—features not replicated in standard in vitro systems. For instance, the elevated presence of M27 and M38 in vivo suggests a potential role for extrahepatic Phase I/II enzymes or tissue-specific biotransformation.

While the primary objective of this study was to characterize the major metabolic routes of R14, we acknowledge the need for further mechanistic investigation to resolve these in vitro–in vivo differences. Future work will include enzyme phenotyping, recombinant CYP assays, and advanced hepatic co-culture models to identify the key metabolic enzymes and clarify the conversion pathways involved in vivo.

Although methylation is conventionally considered a Phase II reaction, our study demonstrated that it can occur in multiple rounds before and after Phase I transformations. This sequential and layered pattern of metabolism reflects the biochemical complexity of drug processing in vivo. The findings emphasize that while in vitro metabolism studies are essential and can offer early insights into a drug’s metabolic profile, they cannot yet fully replace in vivo pharmacokinetic and metabolism studies due to the dynamic and interconnected nature of in vivo enzymatic systems.

In our investigation, we did not perform in vitro glucuronidation and sulfation assays using liver microsomes, due to the absence of a hydroxyl moiety in the parent R14 structure. Nonetheless, conjugated metabolites such as glucuronides and sulfates were identified in both plasma and urine samples, indicating that these modifications occurred following initial hydroxylation. This metabolic sequence—Phase I oxidation or reduction followed by Phase II conjugation—is commonly observed across many xenobiotics and facilitates renal elimination. These results also underscore the importance of evaluating the entire metabolic cascade, especially when investigating structurally similar drug candidates, as bypassing earlier or intermediate reactions may miss key metabolic events.

Extensive metabolism, as seen with R14, also raises potential concerns regarding drug–drug interactions (DDIs). Intermediates such as M1, M27, and M46, although observed as major in vitro metabolites, were rapidly converted in vivo and were either undetectable or present only at trace levels in plasma and urine. This discrepancy illustrates the limitations of in vitro models, which often lack the full complement of enzymatic pathways present in the whole organism. Therefore, comprehensive characterization of metabolic pathways is essential not only to support safe and effective drug development but also to anticipate and mitigate clinically relevant DDIs.

The findings from this study have important implications for the formulation and further development of R14 as a clinical candidate. The identification of extensive metabolism underscores the need for formulation strategies that enhance systemic exposure and metabolic stability. The moderate permeability observed in the Caco-2 assay, combined with the rapid clearance and extensive phase I and phase II biotransformation, suggests that alternative formulation approaches—such as prodrug design, metabolic soft spot modification, or enabling formulations (e.g., nanoemulsions or solid dispersions)—may be necessary to improve oral bioavailability [19,20].

Moreover, the early identification of major metabolites and the CYP isoforms responsible for R14 metabolism provides a foundation for anticipating DDI and optimizing dosing strategies in clinical trials [21,22]. Collectively, these integrated in vitro and in vivo findings contribute to a rational framework for advancing R14 into clinical development with informed decisions on formulation design, safety evaluation, and pharmacokinetic optimization.

Traditionally, metabolite identification has relied on radiolabeled compounds and labor-intensive workflows [23,24,25]. However, recent advances in high-resolution mass spectrometry—particularly QTOF-MS—have significantly enhanced the efficiency and precision of metabolite detection and characterization [26]. QTOF-MS provides molecular mass accuracy up to four decimal places, minimizing mass errors (<2 ppm, as shown in Table 2) and enabling confident structural assignment of unknown metabolites. When integrated with advanced software tools such as MetabolitePilot (used in this study), this technology facilitates rapid and comprehensive metabolite profiling.

Our study employed a holistic approach to characterize the pharmacokinetics and metabolism of R14, including in vivo evaluations following oral administration and complementary in vitro assays involving hepatic enzymes and rat blood. These studies were critical in identifying key metabolic transformations and understanding the systemic exposure of R14 and its metabolites [27,28,29,30,31]. Over recent decades, drug metabolism and pharmacokinetic assessments have been increasingly incorporated into early-phase drug development. The integrated strategy employed in this study proved both efficient and informative, offering essential data to guide further preclinical and clinical development.

Nonetheless, some limitations remain. For instance, the proposed metabolic pathways have not yet been validated using independent orthogonal techniques [32], and the specific positioning of functional groups in some metabolites (e.g., M3, M27, M46) could not be resolved due to intrinsic limitations of MS [26,33,34]. Future study has been planned to incorporate technologies such as liquid chromatography–nuclear magnetic resonance (LC-NMR) to achieve definitive structural confirmation [35,36].

## 5. Conclusions

In summary, we present a comprehensive and integrated roadmap for the preclinical evaluation of a drug candidate. This approach includes assessments of metabolic stability in biological media to support stability-indicating assay development, Caco-2 permeability studies to estimate oral bioavailability, in vitro metabolism using CYP enzymes, liver microsomes, and hepatocytes, as well as in vivo drug metabolism and pharmacokinetic profiling. The use of LC-MS/MS and LC-QTOF MS/MS proved highly efficient and feasible, particularly in early drug discovery.

Specifically, comprehensive in vivo studies successfully characterized the pharmacokinetics and metabolic profile of R14 in rats, identifying 21 plasma and 38 urine metabolites following oral administration. Key metabolites were confirmed, and their metabolic pathways elucidated. In parallel, in vitro studies were conducted to evaluate R14’s permeability, determine the major CYP isoforms involved in its metabolism, and assess the metabolic kinetics of principal in vitro metabolites. This integrated and efficient strategy, combining in vitro and in vivo approaches, provides a streamlined framework for understanding drug disposition and behavior during the early phases of drug development. It serves as a robust model for leveraging modern analytical technologies and strategic study design in support of novel therapeutic advancement.

## Figures and Tables

**Table 1:** PK parameters of R14 after a single dose of R14 to rats (data derived from non-compartmental analysis).

Parameters	MEAN ± SD	
	i.v. Administration (*n* = 3)	Oral Administration (*n* = 3)
Dose (mg/kg)	5	50
C_max_ (ng/mL)	-	1152 ± 201
T_max_ (h)	-	0.5
AUC_0→10h_ (h∙ng/mL)	1582 ± 477	2076 ± 255
AUC_0→inf_ (h∙ng/mL)	1813 ± 444	2426 ± 344
Half-life (h)	5.39 ± 0.94	2.99 ± 1.41
CL(/F) (L/h/kg)	2.63 ± 0.43	22.04 ± 2.18
Vd(/F) (L/kg)	20.70 ± 6.11	93.35 ± 38.08
MRT (h)	1.63 ± 0.39	2.82 ± 0.50
F%	-	13.38
% R14 excreted unchanged in 24 h urine	0.035 ± 0.006	0.021 ± 0.008

**Table 2:** Summary of R14 metabolites. Exact masses, proposed molecular formulas, MS/MS fragments, metabolic reactions and UHPLC retention times (RT).

Metabolite	RT (min)	Formula	Calculated *m*/*z*	Observed *m*/*z*	Mass Error (ppm)	Characteristic Fragment Ions (*m*/*z*)	Reactions	Source			
								U *	P	M	B
R14	19.6	C_14_H_10_FNOS	260.054	260.0539	−0.3	167.9916, 164.0166, 106.0650, 91.0541	Parent				
M1	23.1	C_14_H_12_FNOS	262.0696	262.0695	−0.5	154.9959, 127.0014, 108.0807	Carbonyl reduction		√		√
M2	9.9	C_15_H_12_FNO_3_S	306.0595	306.0598	1.1	276.0493, 185.0072, 169.9836, 288.0493	R14 mono- methylation and di-hydroxylation	√	√		
M3	12.0	C_15_H_14_FNO_3_S	308.0751	308.075	−0.4	185.0062, 169.9829, 122.0165	M1 mono- methylation, di-hydroxylation	√	√		
M4	12.3	C_20_H_20_FNO_7_S	438.1017	438.1017	−0.1	262.0691, 154.9958, 127.0016, 108.0808	M1 glucuronidation	√	√		
M5	13.5	C_15_H_14_FNO_3_S	308.0751	308.0754	0.9	169.0122, 141.0182, 153.9886	M1 mono- methylation, bi-hydroxylation	√	√		
M6	13.7	C_16_H_16_FNO_2_S_2_	338.0679	338.0682	0.8	169.0121, 141.0182	M30 methanethiol addition	√	√		
M7	15.4	C_16_H_16_FNO_2_S_2_	338.0679	338.0683	1.1	169.0121, 141.0172	M30 methanethiol addition	√	√		
M8	20.6	C_15_H_12_FNO_3_S	306.0595	306.0596	0.4	169.0120, 141.0171, 153.9886	R14 mono- methylation and bi-hydroxylation	√	√		
M9	9.5	C_21_H_22_FNO_9_S	484.1072	484.1075	0.6	185.0076, 169.9842	M3 glucuronidation	√	√		
M10	9.8	C_15_H_14_FNO_4_S	324.0700	324.0703	0.8	276.0497, 185.0071, 169.9840	M1 mono-methylation tri-hydroxylation	√			
M11	10.9	C_15_H_14_FNO_4_S	324.0700	324.0703	0.8	201.0027, 185.0047, 306.0603	M1 mono-methylation tri-hydroxylation	√	√		
M12	11.4	C_15_H_14_FNO_6_S_2_	388.0319	388.0319	−0.1	185.0071, 169.9839	M3 sulfation	√			
M13	11.8	C_15_H_14_FNO_2_S	292.0802	292.0804	0.7	169.0121, 153.9885, 141.0172	M1 mono- methylation, mono-hydroxylation	√	√		
M14	11.9	C_21_H_22_FNO_8_S	468.1123	468.1124	0.2	292.0812, 169.0122, 141.0182	M13 glucuronidation	√	√		
M15	12.5	C_21_H_22_FNO_9_S	484.1072	484.1077	1.0	308.0769, 169.0128, 141.0184	M15 glucuronidation	√			
M16	12.6	C_16_H_16_FNO_4_S	338.0857	338.0858	0.3	185.0072, 169.9840, 122.0168	M1 di-methylation tri-hydroxylation	√			
M17	13.2	C_16_H_16_FNO_3_S	322.0908	322.0908	0.1	169.0132, 185.0072, 141.0183	M1 di- methylation, di-hydroxylation	√			
M18	13.5	C_15_H_14_FNO_6_S_2_	388.0319	388.0319	−0.1	169.0127, 141.0175	M5 sulfation	√	√		
M19	13.7	C_22_H_24_FNO_8_S_2_	514.1	514.1002	0.4	338.0689, 169.0123, 141.0184	M6 or M7 glucuronidation	√			
M20	13.9	C_15_H_14_FNO_5_S_2_	372.037	372.0369	−0.3	169.0121, 141.0178	M13 sulfation	√	√		
M21	14.9	C_16_H_16_FNO_3_S	322.0908	322.0908	0.1	169.0129, 141.0170	M1 di- methylation, di-hydroxylation	√	√		
M22	15.3	C_15_H_14_FNO_2_S	292.0802	292.0803	0.3	185.0072, 169.9836	M1 mono- methylation, mono-hydroxylation	√	√		
M23	15.4	C_22_H_24_FNO_8_S_2_	514.1	514.1001	0.2	338.0697, 169.0128, 141.0184	M6 or M7 glucuronidation	√			
M24	7.7	C_15_H_12_FNO_4_S	322.0544	322.0547	1.0	201.0019, 187.0226	R14 mono- methylation and tri-hydroxylation	√			
M25	9.9	C_15_H_14_FNO_7_S_2_	404.0268	404.0271	0.6	185.0066, 169.9833	M10 sulfation	√			
M26	10.3	C_15_H_12_FNO_2_S	290.0646	290.0646	0.2	258.0399, 185.0090, 169.9839, 106.0655, 91.0548	R14 mono- methylation and mono-hydroxylation	√	√		
M27	12.1	C_14_H_10_FNO_2_S	276.0489	276.0490	0.3	106.0652, 79.0545, 77.0387	R14 mono-hydroxylation	√		√	
M28	12.4	C_22_H_24_FNO_9_S	498.1229	498.1232	0.7	169.0128, 322.0924, 141.0181	M21 glucuronidation	√			
M29	12.7	C_15_H_12_FNO_6_S	354.0442	354.0442	0	201.0027, 336.0353, 169.9838	R14 mono- methylation and penta-hydroxylation	√			
M30	13.1	C_15_H_12_FNO_2_S	290.0646	290.0647	0.5	148.0401, 169.0129, 155.0335	R14 mono- methylation and mono-hydroxylation	√	√		
M31	13.7	C_15_H_12_FNO_5_S	338.0493	338.0492	−0.3	185.0076, 169.9843, 122.0171	R14 mono- methylation and tetra-hydroxylation	√			
M32	14.9	C_16_H_16_FNO_6_S_2_	402.0476	402.0479	0.8	169.0120, 141.0176	M21 sulfation	√			
M33	15.5	C_16_H_16_FNO_5_S_3_	418.0247	418.0246	−0.3	169.0127, 141.0178	M6 or M7 sulfation	√			
M34	16.1	C_15_H_12_FNO_5_S	338.0493	338.0497	1.2	201.0029, 169.9844, 320.0410	R14 mono- methylation and tetra-hydroxylation	√	√		
M35	16.6	C_21_H_20_FNO_9_S	482.0916	482.0919	0.7	306.0597, 169.0129, 288.0513	M8 glucuronidation	√			
M36	17.0	C_16_H_16_FNO_5_S_3_	418.0247	418.0246	−0.3	169.0129, 141.0181	M6 or M7 sulfation	√			
M37	17.7	C_15_H_12_FNO_4_S	322.0544	322.0545	0.4	185.0074, 169.9842, 122.0171	R14 mono- methylation and tri-hydroxylation	√			
M38	18.1	C_15_H_14_FNOS	276.0853	276.0856	1.1	169.0129, 153.9898, 141.0188	M1 mono- methylation	√	√		
M39	19.2	C_15_H_12_FNO_4_S	322.0544	322.0548	1.3	169.0128, 141.0178	R14 mono- methylation and tri-hydroxylation	√			
M40	14.9	C_15_H_14_FNO_3_S	308.0751	308.0752	0.2	201.0025, 169.9841	M1 mono- methylation, bi-hydroxylation		√		
M41	8.6	C_14_H_10_FNO_3_S	292.0438	292.0441	1.0	122.0609, 106.0653	R14 di-hydroxylation			√	
M42	9.2	C_14_H_10_FNO_4_S	308.0387	308.0389	0.5	290.0313, 202.9830, 106.0653	R14 tri-hydroxylation			√	
M43	9.6	C_14_H_10_FNO_3_S	292.0438	292.0440	0.6	106.0659	R14 di-hydroxylation			√	
M44	14.0	C_14_H_10_FNO_3_S	292.0438	292.0441	1.0	122.0608, 154.9968	R14 di-hydroxylation			√	
M45	16.7	C_14_H_10_FNO_4_S	308.0387	308.0389	0.5	290.0308, 170.9922, 138.0558	R14 tri-hydroxylation			√	
M46	17.5	C_14_H_10_FNO_2_S	276.0489	276.0492	1.1	122.0607, 154.9973	R14 mono-hydroxylation			√	

*: U = urine; P = plasma; M = phase I metabolism, B = whole blood incubation.

## Data Availability

The data presented in this study are available in this article and Supplementary Materials.

## Supplementary Materials

The following supporting information can be downloaded at: https://www.mdpi.com/article/10.3390/pharmaceutics17081052/s1, Details of partial validation data for the LC-MS/MS method for R14 quantification and details of individual metabolite identification.

## Abbreviations

The following abbreviations are used in this manuscript:

AUC	area under the curve
CAD	collision gas
Cl	clearance
Cl_int_	intrinsic clearance
C_max_	maximum plasma concentration
CE	collision energy
DP	declustering potential
F	oral bioavailability
FDA	The Food and Drug Administration
HR	High resolution
i.v.	intravenous
ICH	the International Council for Harmonisation
IDA	Information-dependent-acquisition
IS	internal standard
K_m_	Michaelis-Menten constant
MRT	mean residence time
MS	mass spectrometry
PBS	phosphate-buffered saline
R14	6-Fluoro-2-(2-methylphenyl)-1,2-benzothiazol-3(2H)-one
RNase	ribonuclease
RT	retention time
SD	Sprague Dawley
T_½_	half-life
TEER	transepithelial electrical resistance
T_max_	time to reach maximum plasma drug concentration
TNBC	triple-negative breast cancer
UHPLC-MS/MS	ultra-high-performance liquid chromatography tandem mass spectrometry
UHPLC-QTOF MS/MS	ultra-high-performance liquid chromatography quadrupole time-of-flight tandem mass spectrometry
V_max_	maximum reaction velocity
V_d_	volume of distribution
