# Supplementary material for: Drug Metabolism and Pharmacokinetic Evaluation of a Novel RNase H2 Inhibitor for the Treatment of Triple-Negative Breast Cancer
Source: Pharmaceutics. 2025 Aug 13;17(8):1052. doi: 10.3390/pharmaceutics17081052 (PMC12389022; doi:10.3390/pharmaceutics17081052)
Supplement: Supplementary file 1 [file pharmaceutics-17-01052-s001.zip › Supplementary Information-3765194.pdf]

Drug metabolism and pharmacokinetic evaluation of a novel RNase H2 inhibitor for the treatment of triple-negative breast cancer

Yang Wang[1], Huan Xie[1], Jing Ma[1], Ting Du[1], Song Gao[1], Yuan Chen[1], Shiaw-Yih Lin[2,*], Dong Liang[1,*]

[1] Department of Pharmaceutical Science, College of Pharmacy and Health Sciences, Texas Southern University, Houston, Texas; yang.wang@tsu.edu (Y.W.); huan.xie@tsu.edu (H.X.); jing.ma@tsu.edu (J.M.); du.ting@tsu.edu (T.D.); song.gao@tsu.edu (S.G.); yuan.chen@tsu.edu (Y.C.);dong.liang@tsu.edu (D.L.)
[2] Department of Systems Biology, MD Anderson Cancer Center, Houston, Texas; sylin@mdanderson.org (S.-Y.L.)
* Correspondence: dong.liang@tsu.edu (D.L.); sylin@mdanderson.org (S.-Y.L.)

Supplementary Information

1. Partial validation data for the LC-MS/MS method for R14 quantification

1.1. Methods

1.1.1 Instrumentation and analytical conditions

R14 quantification via monitoring R14 bisulfite was conducted on a 6500+ Triple Quad LC-MS/MS System (AB SCIEX LLC, CA, USA) coupled with a Synergi Fusion-RP column (50 x 2 mm, 4 μm, 80 Å, Phenomenex Inc., Torrance, CA, USA). Water containing 2 mM ammonium formate and 0.01% FA and acetonitrile were applied as mobile phase A and B. The flow rate and injection volume were set at 0.5 mL and 2 μL, respectively. The time program of the gradient was as follows: initially Phase B was at 30% for 0.5 min, linearly increased from 30% to 80% in 1 min, then stayed at 80% for 0.5 min followed by an increase from 80% to 95% in 0.5 min and stable at 95% for 0.5 min, then decreased from 95% to 30% in 0.5 min and kept stably at 30% for 1 min. Data were acquired using negative multiple reaction monitoring (MRM) mode by Analyst software 1.6.3. The ion spray voltage and temperature were established at -4500 V and 400 °C, respectively. The curtain gas, CAD, gas 1 and gas 2 were set at 45, 9, 50 and

50 psi. The MS/MS parameters for R14 and the internal standard (IS) warfarin are shown in Table S1.

Table S1. Compound dependent parameters for R14 and the IS

	Q1 (m/z) (Da)	Q3 (m/z) (Da)	DP (V)	EP (V)	CE (V)	CXP (V)
R14 bisulfite	340.1	127.0*	-40	-10	-48	-10
		105.9	-40	-10	-45	-10
Warfarin (IS)	307.1	160.9	-55	-10	-28	-10

Q1: precursor ion, Q3: fragment ion, DP: declustering potential, EP: entrance potential, CE: collision energy, CXP: collision cell exit potential.
*: transition *m/z* 340.1 → 127.0 used for R14 quantification.

1.1.2 Quality control (QC) samples

The working solutions for QC samples were independently prepared by diluting the stock solution with acetonitrile and then spiking in blank rat plasma to obtain the following R14 concentrations: 1 (LLOQ), 3 (LQC), 50 (MQC) and 800 ng/mL (HQC).

1.1.3. Method validation

The method was validated according to the US Food and Drug Administration (FDA) Bioanalytical Method Validation Guidelines for Industry.

A calibration curve in the form of y = Ax + B was determined by plotting the peak area ratio of analyte to IS against nominal concentrations of R14. The slope, intercept, and coefficient of determination were estimated using least squares linear regression method with a weighting of 1/x.

The accuracy and precision were determined by replicate analyses of QC samples on one validation day (intra-day n = 6) or on three validation days (inter-day, n = 18). The relative error (RE%) and coefficient of variation (CV%) were used to estimate the accuracy and precision, respectively.

We determined the matrix effect and extraction recovery of R14 at three QC levels. Matrix effects were calculated by comparing the peak area of spiking analyte in the extracted analyte-free blood samples to the peak area in neat solution. Recoveries were expressed as the peak area ratio of QC samples to spiking analyte in the extracted analyte-free blood samples.

1.2. Results

The linearity range of the calibration curves was between 1 to 1000 ng/mL. The regression correlation coefficients were better than 0.99 in all validation runs.

The accuracy and precision results are summarized in Table S2. The intra-day and inter-day accuracy (RE%) were from -6.35 to 4.19% and -5.78 to 3.96%, respectively. The intra-day and inter-day precision (CV%) ranged 5.53 ~ 9.19% and 8.34 ~ 12.44%, respectively. These data indicate that the method is accurate and precise for the quantification of R14 in rat plasma.

Table S2. Intra-day and inter-day accuracy and precision of R14 in rat plasma.

Nominal concentration (ng/mL)	Intra-day (*n*=6)			Inter-day (*n*=18)		
	Observed concentration (mean ± SD)	Accuracy (RE%)	Precision (CV%)	Observed concentration (mean ± SD)	Accuracy (RE%)	Precision (CV%)
1	0.94 ± 0.09	-6.35	9.19	0.94 ± 0.12	-5.78	12.44
3	3.07 ± 0.21	2.33	7.13	2.98 ± 0.29	-0.43	9.75
50	49.42 ± 3.35	-1.17	6.71	49.55 ± 5.44	-1.13	11.47
800	833.50 ± 44.25	4.19	5.53	834.15 ± 65.34	3.96	8.34

Mean matrix effect and recovery were 95.41 ± 5.31 and 94.59 ± 4.98 % at 3 QC levels, meaning the matrix effect was negligible and the extraction was thorough in this study (Table S3).

Table S3. Recovery and matrix effect of R14.

Nominal concentration (ng/mL)	Matrix effect (%) (*n*=6)	Recovery (%) (*n*=6)
3	93.36 ± 7.40	99.65 ± 5.99
50	94.89 ± 5.71	92.37 ± 1.62
800	97.98 ± 1.06	91.77 ± 1.14

2. Individual metabolite identification

Individual metabolite identification and confirmation process were described below:

Metabolites M2 and M8: M2 and M8 with protonated ions at m/z 306.0598 and 306.0596 were eluted at 9.9 and 20.8 min on the UHPLC-MS/MS in the urine sample, respectively. Both M2 and M8 have an identical proposed formula $C_{15}H_{12}FNO_3S$ (calculated $[M+H]^+$ at m/z 306.0597, mass errors 1.1 and 0.4 ppm), which had an additional component of CH_2O_2 from R14, suggesting mono-methylation and di-hydroxylation products of R14. MS/MS spectra of M2 achieved characterized ions at m/z 276.0493 ($[M+H-CH_2-O]^+$ calculated m/z 276.0489), 185.0072, and 169.9836 ($[OHFC_6HCOS+H]^+$ calculated m/z 169.9832), suggesting mono-hydroxylation and mono-methylation occurred on Ring A, and the other hydroxylation should be on Ring C. The MS/MS spectra of M8 achieved characterized ions at m/z 169.0120 and 141.0171 ($[CH_3FC_6H_2S+H]^+$ calculated m/z 141.0169), which were the characterized ions of mono-methylation on Ring A, and the di-hydroxyl groups attach to Ring C.

Metabolites M5 and M40: M5 and M40 with protonated ions at m/z 308.0754 and 308.0752 were identified at 13.5 min in the urine samples and 14.9 min in the plasma samples on the UHPLC-MS/MS, respectively. M5 and M40 had the same proposed formula of $C_{15}H_{14}FNO_3S$ (calculated $[M+H]^+$ at m/z 308.0751, mass errors 0.9 and 0.2

ppm), suggesting mono-methylation and di-hydroxylation products of M1. In MS/MS spectra, the specific fragments of M5 were ions at m/z 169.0122 and 141.0182, implying mono-methylation on Ring A, and di-hydroxylation on Ring C. M40 was identified from the plasma samples with specific fragments at m/z 201.0025 and 169.9841, suggesting that all substitutions (mono-methylation and di-hydroxylation) were on Ring A.

Metabolites M6 and M7: M6 and M7 with protonated ions at m/z 338.0682 and 338.0683 were identified at 13.7 and 15.4 min, respectively, on the UHPLC-MS/MS from the rat urine samples. Both M6 and M7 had the same proposed formula $C_{16}H_{16}FNO_2S_2$ (calculated $[M+H]^+$ at m/z 338.0679, mass errors 0.8 and 1.1 ppm), which had an additional CH_3SH moiety compared to M26 and M30 (mono-methylation and mono-hydroxylation products of R14), respectively, suggesting a methanethiol addition of M26 and M30. The methanethiol addition was most likely to occur at the carbonyl group. The MS/MS spectra of M6 and M7 achieved similar major product ions at m/z 169.0121 and 141.0182; and 169.0121 and 141.0172, respectively, which were characteristic fragments of mono-methylation on ring A, and the hydroxylation potentially on ring C. Therefore, M6 and M7 were proposed as the methanethiol addition products of M30.

Metabolites M9 and M15: M9 and M15 with protonated ions at m/z 484.1075 and 484.1077 were eluted at 9.5 and 12.5 min, respectively, on the UHPLC-MS/MS from the rat urine samples. Both M9 and M15 had the same proposed formula $C_{21}H_{22}FNO_9S$ (calculated $[M+H]^+$ at m/z 484.1072, mass errors 0.6 and 1.0 ppm), which had an additional component of $C_6H_8O_6$ from M3 and M5, respectively, implying glucuronide conjugates of M3 and M5. The MS/MS spectra of M9 provided characteristic product

ions at m/z 185.0076 and 169.9842, implying mono-methylation and mono-hydroxylation on Ring A. The protonated ion of M15 formed fragments at m/z 308.0769, 169.0128, and 141.0184, suggesting mono-methylation on Ring A. Thus, M9 was the glucuronide product of M3, and M15 was the glucuronide product of M5.

Metabolites M10 and M11: M10 and M11 with protonated ions at m/z 324.0703 were eluted at 9.8 and 10.9 min, respectively, on the UHPLC-MS/MS from the rat urine samples. Both M10 and M11 had the same proposed formula $C_{15}H_{14}FNO_4S$ (calculated $[M+H]^+$ at m/z 324.0700, mass errors 0.8 ppm), which had an additional component of CH_2O_3 from M1, implying mono-methylation and tri-hydroxylation products of M1. The MS/MS spectra of M10 showed major product ions at m/z 185.0071 and 169.9840, implying mono-methylation and mono-hydroxylation on Ring A and another di-hydroxylation proposed on Ring C. M10 could be produced via hydroxylation of either M3 (on Ring C) and/or M5 (on ring A). The MS/MS spectra of M11 showed major product ions at m/z 201.0027 and 185.0047, implying mono-methylation and di-hydroxylation on ring A and another hydroxylation on Ring C.

Metabolites M12 and M18: M12 and M18 with protonated ions at m/z 388.0319 were eluted at 11.4 and 13.5 min, respectively, on the UHPLC-MS/MS from the rat urine samples. Both M12 and M18 had the same proposed formula $C_{15}H_{14}FNO_6S_2$ (calculated $[M+H]^+$ at m/z 388.0319, mass errors -0.1 ppm), which had an additional component of SO_3 from M3 and M5, respectively, implying the sulfation products of M3 and M5. The MS/MS spectra of M12 achieved characteristic product ions at m/z 185.0071 and 169.9839, indicating mono-hydroxylation and mono-methylation on Ring A, therefore M12 was the sulfate conjugate of M3. The MS/MS spectra of M18 achieved

characteristic product ions at m/z 169.0127 and 141.0175, indicating mono-methylation on Ring A, therefore M18 was the sulfate conjugate of M5.

Metabolites M13 and M22: M13 and M22 with protonated ions at m/z 292.0804 and 292.0803 were eluted at 11.9 and 13.9 min, respectively, on the UHPLC-MS/MS from the rat urine samples. Both M13 and M22 had the same proposed formula $C_{15}H_{14}FNO_2S$ (calculated $[M+H]^+$ at m/z 292.0802, mass errors 0.7 and 0.3 ppm), which had an additional component of CH_2O from M1, implying mono-methylation and mono-hydroxylation products of M1. The MS/MS spectra of M13 achieved characteristic product ions at m/z 169.0121, 153.9885 and 141.0172, indicating mono-methylation on Ring A and hydroxylation on Ring C. The MS/MS spectra of M22 showed major product ions at m/z 185.0072 and 169.9836, implying both the methylation and hydroxylation happened on Ring A.

Metabolite M14: M14 with a protonated ion at m/z 468.1124 was eluted at 11.8 min on the UHPLC-MS/MS from the rat urine samples. M14 had a proposed formula $C_{21}H_{22}FNO_8S$ (calculated $[M+H]^+$ at m/z 468.1123, mass error 0.2 ppm), which had an additional component of $C_6H_8O_6$ from either M13 or M22, implying a glucuronide conjugate of M13 or M22. The MS/MS spectra of M14 showed major product ions at m/z 292.0812, 169.0122, and 141.0182, indicating mono-methylation on ring A. Thus, M14 was the glucuronide conjugate of M13.

Metabolite M16: M16 with a protonated ion at m/z 338.0858 was eluted at 12.6 min on the UHPLC-MS/MS from the rat urine samples. M16 had a proposed formula $C_{16}H_{16}FNO_4S$ (calculated $[M+H]^+$ at m/z 338.0857, mass error 0.3 ppm), which (a) had an additional component of $C_2H_4O_3$ from M1, implying di-methylation and tri-

hydroxylation of M1; (b) had 16 Da more than M17 and M21, implying mono-hydroxylation product of M17 and M21; and (c) had an additional component of CH_2 from M10 and M11, suggesting mono-methylation product of M10 or M11. The MS/MS fragmentation of M16 achieved characterized ions at m/z 185.0072 and 169.9840, indicating mono-hydroxylation and mono-methylation on Ring A, and the other mono-methylation and di-hydroxylation on Ring C. Thus, M16 might be a hydroxylation product of M21 on ring A, or a hydroxylation product of M17 on ring C, or methylation product of M10 on Ring C.

<u>Metabolites M17 and M21:</u> M17 and M21 with protonated ions at m/z 322.0908 were eluted at 13.2 and 14.9 min, respectively, on the UHPLC-MS/MS from the rat urine samples. Both M17 and M21 had the same proposed formula $C_{16}H_{16}FNO_3S$ (calculated $[M+H]^+$ at m/z 322.0908, mass errors 0.1 ppm), which had an additional component of (a) $C_2H_4O_2$ from M1 (suggesting di-methylation and di-hydroxylation products of M1); (b) CH_2O from M13 and M22 (suggesting mono-methylation and mono-hydroxylation products of M13 and M22); and (c) CH_2 from M3 and M5 (suggesting mono-methylation products of M3 and M5). The MS/MS spectra of M17 provided characteristic product ions at m/z 185.0072 and 169.0132, indicating mono-methylation and mono-hydroxylation on Ring A. The other mono-methylation and mono-hydroxylation would have to be on Ring C. The MS/MS spectra of M21 provided characteristic product ions at m/z 169.0129 and 141.0170, indicating mono-methylation on Ring A. The other mono-methylation and di-hydroxylation would have to be on Ring C.

<u>Metabolites M19 and M23:</u> M19 and M23 with protonated ions at m/z 514.1002 and 514.1001 were eluted at 13.7 and 15.4 min, respectively, on the UHPLC-MS/MS

from the rat urine samples. Both M19 and M23 had the proposed formula $C_{22}H_{24}FNO_8S_2$ (calculated $[M+H]^+$ at m/z 514.1000, mass errors 0.4 and 0.2 ppm), which had an additional component of $C_6H_8O_6$ from M6 or M7, implying glucuronide conjugates of M6 and M7, respectively. The MS/MS spectra provided characteristic product ions at m/z 338.0689, 169.0123, and 141.0184 for M19, and 338.0697, 169.0128, and 141.0184 for M23, further confirming M19 and M23 were glucuronide conjugates of M6 and M7, respectively.

Metabolite M20: M20 with a protonated ion at m/z 372.0369 was eluted at 13.9 min on the UHPLC-MS/MS from the rat urine samples. M20 had a proposed formula $C_{15}H_{14}FNO_5S_2$ (calculated $[M+H]^+$ at m/z 372.0370, mass error -0.3 ppm), which had an additional component of SO_3 from M13 or M22, implying a sulfation product of M13 or M22. The MS/MS spectra of M20 provided characteristic product ions at m/z 169.0121 and 141.0178, indicating mono-methyl group on Ring C. Therefore, M20 was considered a sulfate product of M13.

Metabolites M24 and M37, M39: M24, M37 and M39 with protonated ions at m/z 322.0547, 322.0545, and 322.0548 were eluted at 7.7, 17.7 and 19.2 min, respectively, on the UHPLC-MS/MS from the rat urine samples. All three had the same proposed formula $C_{15}H_{12}FNO_4S$ (calculated $[M+H]^+$ at m/z 322.0544, mass errors 1.0, 0.4 and 1.3 ppm), which was 16 Da more than M2 or M8, implying mono-hydroxylation products of M2 or M8. The MS/MS spectra of M24 provided characteristic product ions at m/z 201.0019 and 187.0226, indicating mono-methyl group and di-hydroxyl groups on Ring A, and an additional mono-hydroxyl group on Ring C, suggesting M24 was a hydroxylation product of M2 on ring A. The MS/MS spectra of M37 provided

characteristic product ions at m/z 185.0074 and 169.9842, indicating mono-methyl group and mono-hydroxyl group on Ring A, and di-hydroxyl groups on Ring C, suggesting M37 was either a hydroxylation product of M2 on Ring C, or a hydroxylation product of M8 on Ring A. The MS/MS spectra of M39 provided characteristic product ions at m/z 169.0128 and 141.0178, indicating mono-methyl group on Ring A, and then tri-hydroxyl groups on Ring C, suggesting M39 was a hydroxylation product of M8 on Ring C.

Metabolite M25: M25 with a protonated ion at m/z 404.0271 was eluted at 9.9 min on the UHPLC-MS/MS from the rat urine samples. M25 had a proposed formula $C_{15}H_{14}FNO_7S_2$ (calculated $[M+H]^+$ at m/z 404.0268, mass error 0.6 ppm), which had an additional component of SO_3 from M10 or M11, implying a sulfate product of M10 or M11. The MS/MS spectra of M25 provided characteristic product ions at m/z 185.0066 and 169.9833, indicating mono-methyl group and mono-hydroxyl group on Ring A, and the di-hydroxylation and sulfation on Ring C. Therefore, M25 was a sulfate conjugate of M10.

Metabolites M26 and M30: M26 and M30 with protonated ions at m/z 290.0646 and 290.0647 were eluted at 10.3 and 13.1 min, respectively, on the UHPLC-MS/MS from the rat urine samples. Both M26 and M30 had the same proposed formula $C_{15}H_{12}FNO_2S$ (calculated $[M+H]^+$ at m/z 290.0646, mass errors 0.2 and 0.5 ppm), which had an additional component of CH_2O from R14, implying mono-methylation and mono-hydroxylation products of R14. The MS/MS spectra of M26 provided characteristic product ions at m/z 258.0399 ($[M-CH_3OH+H]^+$ calculated m/z 258.0383), 185.0090, 169.9839 and 106.0655, indicating both methylation and hydroxylation on Ring A. The

MS/MS spectra of M30 provided characteristic product ions at m/z 169.0129, 148.0401, and 155.0335, indicating mono-methylation on Ring A and mono-hydroxylation on Ring C.

Metabolites M27 and M46: M27 and M46, with protonated ions at m/z 276.0490 and 276.0492, respectively, were eluted at 12.1 and 17.5 min, respectively, on the UHPLC-MS/MS from the phase I metabolic reaction samples. M27 and M46 had the same proposed formula $C_{14}H_{10}FNO_2S$, corresponding to a calculated $[M+H]^+$ at m/z 276.0489, with mass errors 0.3 and 1.1 ppm. This represented a 16 Da addition to R14, suggesting that M27 and M46 were hydroxylation products of R14. The MS/MS spectrum of M27 exhibited characteristic product ions at m/z 106.0652 and 79.0545, indicating that no substitution occurred on Ring C and instead mono-hydroxylation on Ring A. In contrast, the MS/MS spectrum of M46 showed characteristic product ions at m/z 122.0607 and 154.9973, confirming that no substitution occurred on Ring A and instead mono-hydroxylation occurred on Ring C. In addition, M27 was detected in both urine and the *in vitro* phase I hepatic microsomal metabolic reaction, whereas M46 was identified only in the phase I metabolic reaction. Both metabolites were major products of R14 phase I metabolism.

Metabolite M28: M28 with a protonated ion at m/z 498.1232 was eluted at 12.4 min on the UHPLC-MS/MS from the rat urine samples. M28 had a proposed formula $C_{22}H_{24}FNO_9S$ (calculated $[M+H]^+$ at m/z 498.1229), which had an additional component of $C_6H_8O_6$ from M17 or M21, implying a glucuronide conjugate of M17 or M21. The MS/MS spectra of M28 provided characteristic product ions at m/z 322.0924, 169.0128,

and 141.0181, indicating mono-methyl group on Ring A. Thus, M28 was a glucuronide conjugate of M21.

Metabolites M31 and M34: M31 and M34 with protonated ions at m/z 338.0492 and 338.0497 were eluted at 13.7 and 16.1 min, respectively, on the UHPLC-MS/MS from the rat urine samples. Both M31 and M34 had the same proposed formula $C_{15}H_{12}FNO_5S$ (calculated $[M+H]^+$ at m/z 338.0493, mass errors -0.3 and 1.2 ppm), which had an additional component of CH_2O_4 from R14, suggesting it was mono-methylation and tetra-hydroxylation products of R14, or mono-hydroxylation products of M24, M37, or M39. The MS/MS fragmentation of M31 achieved characterized ions at m/z 185.0076 and 169.9843, indicating mono-methyl group and mono-hydroxyl group on Ring A. Thus, M31 was a hydroxylation product of M39. The MS/MS fragmentation of M34 achieved characterized ions at m/z 201.0029 and 169.9844, indicating mono-methyl group and di-hydroxyl groups on Ring A. Thus, M34 was a hydroxylation product of M24.

Metabolite M32: M32 with a protonated ion at m/z 402.0479 was eluted at 14.9 min on the UHPLC-MS/MS from the rat urine samples. M32 had a proposed formula $C_{16}H_{16}FNO_6S_2$ (calculated $[M+H]^+$ at m/z 402.0476, mass error 0.8 ppm), which had an additional component of SO_3 from M17 or M21, implying sulfation conjugate of 17 or M21. The MS/MS fragmentation of M32 achieved characterized ions at m/z 169.0120 and 141.0176, indicating mono-methylation on Ring A. Thus, M32 was a sulfate conjugate of M21.

Metabolites M33 and M36: M33 and M36 with protonated ions at m/z 418.0246 were eluted at 15.5 and 17 min, respectively, on the UHPLC-MS/MS from the rat urine

samples. Both M33 and M36 had the same proposed formula $C_{16}H_{16}FNO_5S_3$ (calculated $[M+H]^+$ at m/z 418.0247, mass errors -0.3 ppm), which had an additional component of SO_3 from M6 or M7, implying sulfation conjugates of M6 or M7. The MS/MS spectra provided characteristic product ions at m/z 169.0127 and 141.0178 for M33, and 169.0129 and 141.0181 for M36, indicating mono-methylation on Ring A, which was consistent with M6 and M7, respectively. Thus, M33 and M36 were tentatively confirmed as the sulfate products of M6 and M7, respectively.

Metabolite M35: M35 with a protonated ion at m/z 482.0919 was eluted at 16.6 min on the UHPLC-MS/MS from the rat urine samples. M35 had a proposed formula $C_{21}H_{20}FNO_9S$ (calculated $[M+H]^+$ at m/z 482.0916, mass errors 0.7 ppm), which had an additional component of $C_6H_8O_6$ from M2 or M8, implying a glucuronide conjugate of M2 or M8. The MS/MS spectra of M35 provided characteristic product ions at m/z 306.0597 ($[C_{15}H_{12}FNO_3S+H]^+$ calculated m/z 306.0595), 169.0129, and 288.0513 ($[C_{15}H_{12}FNO_3S-H_2O+H]^+$ calculated m/z 288.0489), indicating mono-methylation on Ring A. Thus, M35 was a glucuronide conjugate of M8.

Metabolite M38: M38 with a protonated ion at m/z 276.0856 was eluted at 18.1 min on the UHPLC-MS/MS from the rat urine samples. M38 had a proposed formula $C_{15}H_{14}FNOS$ (calculated $[M+H]^+$ at m/z 276.0853, mass errors 1.1 ppm), which had an additional component of CH_2 from M1, implying a mono-methylation product of M1. The MS/MS spectra of M38 showed major fragment ions at m/z 169.0129 and 141.0188, indicating the methyl substitution occurred on Ring A. Thus, M38 was a methylation product of M1.

Metabolites M41, M43 and M44: M41, M43, and M44 with protonated ions at m/z 292.0441, 292.0440, and 292.0441 were eluted at 8.6, 9.6 and 14.0 min, respectively, on the UHPLC-MS/MS from the phase I metabolic reaction samples. All three metabolites had the same proposed formula $C_{14}H_{10}FNO_3S$ (calculated $[M+H]^+$ at m/z 292.0438, mass errors 1.0, 0.6, and 1.0 ppm), which had an additional component of O_2 from R14, implying di-hydroxylation products of R14. The MS/MS spectra of M41 and M44 achieved characteristic product ions at m/z 122.0609 and 122.0608, respectively, suggesting that M41 and M44 had mono-hydroxylation on Ring C, and the other hydroxyl group substituted on ring A, but exact positions of the hydroxylation were yet to be determined. The MS/MS spectra of M43 achieved major ion at m/z 106.0659, implying no hydroxylation on Ring C. Thus, M44 was a di-hydroxylation of R14 on Ring A.

Metabolites M42 and M45: M42 and M45 with protonated ions at m/z 308.0389 were eluted at 9.2 and 16.7 min, respectively, on the UHPLC-MS/MS from the phase I metabolic reaction samples. Both M42 and M45 had the same proposed formula $C_{14}H_{10}FNO_4S$ (calculated $[M+H]^+$ at m/z 308.0387), which had an additional component of O_3 from R14, implying triple hydroxylation products of R14. The MS/MS spectra of M42 achieved similarly characteristic product ions at m/z 290.0313 ($[M-H_2O+H]^+$ calculated m/z 290.0282), 202.9830 ($[FC_6(OH)_3COS+H]^+$, calculated m/z 202.9809, three hydroxylation on ring A), and 106.0653 (no substitution on Ring C). Thus, M42 was a tri-hydroxylation metabolite of R14 on Ring A. The MS/MS spectra of M45 achieved characteristic product ions at m/z 290.0308, 262.0344 ($[M-H_2O-CO+H]^+$ calculated m/z 262.0333), 198.0730 ($[NC_6H_2CH_3(OH)_2CO+CH_3OH+H]^+$, calculated m/z

198.0761), 170.9922 ($[OHFC_6H_2COS+H]^+$ calculated m/z 170.9911, mono-hydroxylation on Ring A), 122.0605, and 138.0558 ($[NC_6H_2CH_3(OH)_2+H]^+$ calculated m/z 138.0550, di-hydroxylation on Ring C). The evidence implied that M45 was a R14 metabolite with mono-hydroxyl group on ring A and di-hydroxyl groups on Ring A.
